# Postoperative Diet with an Oligomeric Hyperproteic Normocaloric Supplement versus a Supplement with Immunonutrients in Colorectal Cancer Surgery: Results of a Multicenter, Double-Blind, Randomized Clinical Trial

**DOI:** 10.3390/nu14153062

**Published:** 2022-07-26

**Authors:** Jorge Alejandro Benavides-Buleje, Pedro Vicente Fernández-Fernández, Elena Ruiz-Úcar, Amparo Solana-Bueno, Pedro Antonio Parra-Baños, Beatriz Martínez-Torres, Roberto Lozoya-Trujillo, María Dolores Ruiz-Carmona, Marina Alarcón-Iranzo, Lorena Rentero-Redondo, Emilio Peña-Ros, José Manuel Muñoz-Camarena, Milagros Carrasco-Prats, María Ramírez-Faraco, Paloma Portillo-Ortega, Antonio Albarracín-Marín-Blázquez

**Affiliations:** 1 Coloproctology Unit, Department of Surgery, Reina Sofia University Hospital, 30003 Murcia, Spain; pedrov.fernandez@gmail.com (P.V.F.-F.); pedroapb@yahoo.es (P.A.P.-B.); emilio.doctor@gmail.com (E.P.-R.); jose.camarena61@gmail.com (J.M.M.-C.); carrascoprats@gmail.com (M.C.-P.); mramirezfaraco@yahoo.es (M.R.-F.); antonio.albarracinmb@gmail.com (A.A.-M.-B.); 2 Department of Surgery, University Hospital of Fuenlabrada, 28942 Madrid, Spain; eruizucar@gmail.com (E.R.-Ú.); bmartinezt@salud.madrid.org (B.M.-T.); 3 Department of Surgery, Hospital of Sagunto, 46520 Valencia, Spain; solana.amp@gmail.com (A.S.-B.); rolosurg@hotmail.com (R.L.-T.); ruiz.lola@hotmail.com (M.D.R.-C.); marina.alarcon.iranzo.88@gmail.com (M.A.-I.); 4 Department of Pharmacy, Reina Sofia University Hospital, 30003 Murcia, Spain; lorenarentero13@hotmail.com; 5 Nutrition Unit, Reina Sofia University Hospital, 30003 Murcia, Spain; paloma80es@hotmail.com

**Keywords:** diet, dietary supplements, nutrients, nutritional status, immunity, malnutrition, gastrointestinal neoplasms, perioperative care, postoperative complications

## Abstract

(1) Background: For normo-nourished colorectal cancer patients, the need for immunonutrients after elective surgery is not known. (2) Methods: Multicenter, randomized, double-blind, phase III clinical trial comparing the postoperative diet with 200 mL oligomeric hyperproteic normocaloric (OHN; experimental arm) supplement vs. 200 mL immunonutritional (IN) (active comparator) supplement twice a day for five days in 151 normo-nourished adult colorectal-resection patients following the multimodal rehabilitation ERAS protocol. The proportions of patients with complications (primary outcome) and those who were readmitted, hospitalized for <7 days, had surgical site infections, or died due to surgical complications (secondary outcome) were compared between the two groups until postoperative day 30. Tolerance to both types of supplement and blood parameters was also assessed until day 5. (3) Results: Mean age was 69.2 and 84 (58.7%) were men. Complications were reported in 41 (28.7%) patients and the incidence did not differ between groups (18 (25%) vs. 23 (32.4%) patients with OHN and IN supplement, respectively; *p* = 0.328). No significant differences were found for the rest of the variables. (4) Conclusions: IN supplement may not be necessary for the postoperative recovery of colorectal cancer patients under the ERAS regimen and with normal nutritional status at the time of surgery.

## 1. Introduction

Malnutrition plays a key role in the occurrence of postoperative complications by hindering immune response mechanisms and modifying the inflammatory response. Specifically, anabolic and tissue regeneration processes and immune response to fight infection may be altered [1]. Thus, infection and malnutrition are closely related and may induce or enhance each other.

Immunonutrients are specific nutrients or pharmaconutrients with immunomodulatory properties. In addition to regulating the host’s immune response, they help maintain the mucosal barrier function and adjust local and systemic inflammation, nitrogen levels and protein synthesis [2]. In this regard, most nutritional interventions including immunonutritional (IN) supplement have been carried out in the context of multimodal rehabilitation (MMRH) programs (enhanced recovery after surgery (ERAS) protocol) for colorectal cancer surgery [3]. However, whether immunonutrition may be advantageous in minimizing postoperative morbidity in all patients requiring surgery (and especially those presenting gastrointestinal neoplasms) is an ongoing debate [4,5].

Overall, optimal IN supplement dosage and treatment duration are not well established for patients undergoing surgery [6]. Recent data may support the administration of immunonutrients at the presurgical stage (i.e., 5–7 days before the procedure) [2,7,8]. Postoperatively, however, IN supplementation may increase morbidity in critically ill patients [9]. In fact, maintaining or starting it after surgery is only recommended for malnourished patients for 5–7 days or until they can resume oral feeding that covers at least 60% of their needs [2]. In connection with this, one of the main recommendations of the European Society for Clinical Nutrition and Metabolism (ESPEN) guidelines for surgical patients is the early start of post-surgical nutrition (24–48 h after the intervention) to reduce complications, infections, and days of stay until an optimal nutritional status is recovered [10]. However, nutrient intake may be challenged by the high levels of postsurgical stress in response to invasive surgery. This, in turn, causes a drop in the intestinal absorptive capacity and gastric motility and slows down gastric emptying, among other consequences, which also questions the administration of immune modulators via enteral feeding [11].

For normo-nourished patients and/or those without any nutritional risks, IN supplementation after surgery may not be necessary, although it is a common clinical practice. Instead, the main objective would be to maintain the good nutritional status to ensure the absorption of nutrients and overall digestive tolerance, which may lead to better recoveries after surgery and reduce complications. To help clarify this, our objective was to compare the therapeutic efficacy and safety of a postoperative diet with an oligomeric hyperproteic normocaloric (OHN) supplement and another one containing immunonutrients in patients with colon cancer and normal baseline nutritional status.

## 2. Materials and Methods

### 2.1. Study Design

The NUTRICOLON study was a multicenter, randomized, double-blind, active-controlled, parallel-group, phase III clinical trial with colon cancer adult patients subject to colorectal surgery and following the multimodal ERAS fast-track regimen. The study was performed at the General Surgery and Digestive Diseases Services of the Reina Sofía University General Hospital (HGURS) (Murcia, Spain), Sagunto Hospital (Valencia, Spain), and University Hospital of Fuenlabrada (Madrid, Spain). The study was registered at ClinicalTrials.gov (Identifier: NCT04059731). This report adheres to the Consolidated Standards of Reporting Trials (CONSORT) 2010 statement [12].

### 2.2. Study Participants

The study participants of the NUTRICOLON study were adults with colorectal adenocarcinoma scheduled for oncologic surgery. Inclusion criteria were being 18 or older, having a presurgical clinical diagnosis of stage I-III colorectal carcinoma [13,14], with normal nutritional status (or without the need for any nutritional interventions) and a Malnutrition Screening Tool (MST) score < 2 [15], treated according to ERAS protocol for patients with intestinal anastomosis [16], and having signed the informed consent. Exclusion criteria were being on American Society of Anesthesiologists (ASA) stage IV [17], having chronic renal failure requiring dialysis, being pregnant, and having difficulty understanding the purpose of the study and monitoring.

Patient data were collected by the surgeon and members of the study by using a Case Report Form (CRF), anonymized and uploaded to a specially designed database, to which only a designated statistician who was not part of the study had access.

All subjects provided their written informed consent to participate in the study. The ethics committee of HGURS (Murcia, Spain) approved the study, which complied with the Declaration of Helsinki. Patient recruitment began in May 2019, with inclusion completion scheduled for May 2020. However, due to the COVID-19 pandemic, the inclusion period was extended to December 2020. The last patient data entry was in January 2021.

### 2.3. Interventions

All patients were assessed in the outpatient clinic prior to the intervention (first visit; diagnosis) and 30 days after the surgery (second visit; follow up). During the first visit, patients were referred to a nutritionist consultation. A blood test and the MST scale were used to assess their nutritional status and, if necessary, apply the corresponding nutrition therapy to reach surgery with a normonourished status (MST score < 2).

After the surgery, eligible patients received a postoperative diet supplemented with 200 mL OHN containing 4 g protein/100 mL, no fiber and low lipid content (Survimed^®^ OPD Drink, Fresenius Kabi, Barcelona, Spain; experimental arm) or 200 mL hyperproteic (8 g/100 mL) hypercaloric IN supplementation with fiber and high lipid content (Impact^®^, Nestlé Health Science, Barcelona, Spain or Atempero^®^, Vegenat, Badajoz, Spain; active comparator arm) and oral supplement twice a day for five days.

### 2.4. Variable Description and Outcome Measures

The 30-day postoperative complications were monitored and ranked according to the Clavien-Dindo classification [18]. Surgical site infections [19], the length of hospital stays, and the number of hospital readmissions and deaths associated with the surgery were also recorded during a 30-day postoperative period. The following blood parameters were evaluated preoperatively (first visit to the outpatient clinic and on the day of the surgery) and on the fifth postoperative day: Hemoglobin, leukocytes, lymphocytes, procalcitonin, and C-reactive protein (analytical) and total protein, albumin, prealbumin, transferrin, and creatinine levels (nutritional) [7]. Blood parameters analyzed at hospital admission were used to apply the MST < 2 eligibility criterion, i.e., having normal nutritional status. Oral tolerance to dietary supplement was assessed postoperatively and until day 5. The levels of tolerance were related to the daily dose consumption: Complete (400 mL), partial (200 mL) and no (<200 mL) tolerance.

The primary endpoint was the therapeutic efficacy of the postoperative OHN vs. the IN supplement in patients under ERAS regimen for colon cancer surgery and with normal nutritional status (MST < 2) before the procedure. The outcome measure was the proportion of subjects in each study arm with any postoperative complications within 30 days after the surgery. The secondary endpoint was the therapeutic safety of OHN vs. the IN postoperative supplement. It was analyzed by the proportion of patients in each study group who were readmitted, stayed at the hospital for seven days or less, died due to postoperative complications, and had surgical site infections within 30 days after the surgery. We also compared the tolerance to both types of oral supplement and analyzed blood test results until postoperative day 5.

### 2.5. Sample Size

Sample size calculations were based on the previous multicenter randomized trial conducted in Spain and published by Moya et al. [7]. The authors found that 77% of colorectal cancer patients under immunonutrient supplemented fast-track regimen had no complications postoperatively. Thus, we considered a success rate of 77% for the active comparator arm (IN) and a non-inferior success rate of 86% for the experimental treatment (OHN). Then, the number of patients was calculated assuming 80% of statistical power to reject the null hypothesis (difference of proportions of OHN (P1) and IN (P2) supplement-related parameters is smaller than the non-inferiority limit, i.e., P1-P2 < 0.1), a 0.5 significance level and a 1:2 patient ratio between the IN and OHN groups. As a result, 51 patients were expected for each study arm, although this number increased to 54 patients per group (108 patients in total) when 5% of the drop-out events were considered.

### 2.6. Randomization and Blinding

Subjects were randomly allocated in a 1:1 ratio by the six-cell balanced block randomization method, which was supervised by a designated person outside the trial and from the hospital pharmacy service. Investigators enrolling participants, collecting patient data, and administering intervention as well as study subjects were blinded to participant allocation (double-blind). The supervisor at the pharmacy service also provided the investigators with the corresponding dietary supplement according to the randomization list. The study medication was filled into 200 mL tinted (amber) plastic bottles (code 225433) and numbered and labelled with the study name “NUTRICOLON”.

### 2.7. Statistical Methods

Absolute and relative frequencies were used to describe qualitative variables for both study groups. Quantitative variables were described by the mean and standard deviation (SD) or the median and range. For qualitative variables, group comparisons were analyzed with Chi-squared or Fisher’s tests. We also used Bonferroni corrections for column comparisons. For quantitative variables, Student’s *t*-tests were applied upon verifying normality (Kolmogorov–Smirnov tests) and homogeneity (Levene tests) of variances. All statistical comparisons were made using a two-sided test with a significance level of *p* < 0.05. For the non-inferiority analysis of variables, the Farrington–Manning (F-M) Score and Miettinen–Nurminen (M-N) Score tests were used, with a significance level of 0.05 and a non-inferiority limit of 0.10.

IBM’s Statistical Package for the Social Sciences (SPSS^®^) for Windows version 24 was used for the general statistical analysis. The non-inferiority analysis was performed with R Version 3.0.0.

The primary analysis of the study was based on the Intent-to-Treat population.

## 3. Results

### 3.1. Patient Recruitment and Baseline Characteristics

**Figure 1:**
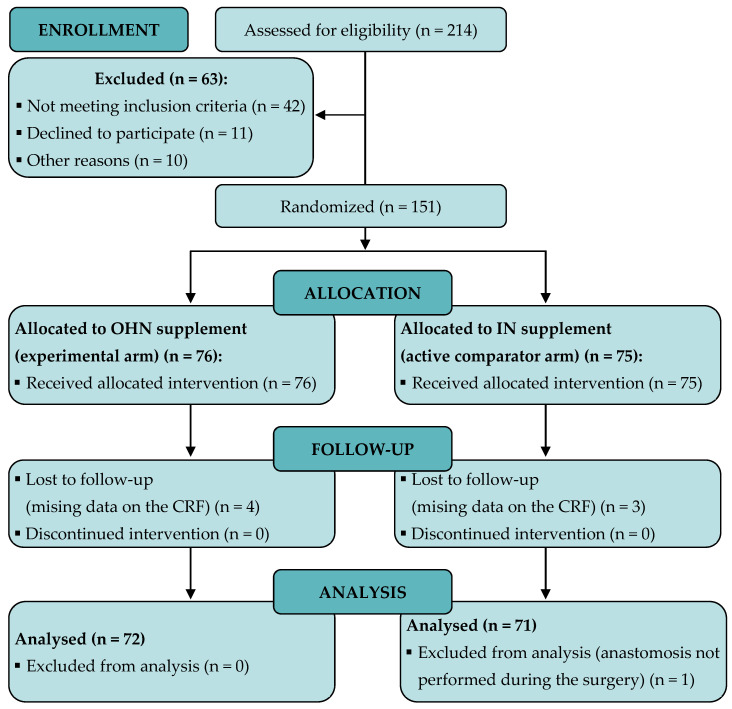
Flow chart of study participants. IN: Immunonutritional; OHN: Oligomeric hyperproteic normocaloric.

A total of 214 patients were assessed for eligibility. Forty-two did not meet the inclusion criteria. Of them, nine had not reached normal nutrition status prior to the surgery. Another twenty-one patients did not agree to participate or were excluded for other reasons. Then, 151 patients were randomized and 143 patients had data for the study analysis (Figure 1).

The mean age of study population was 69.2 (SD = 11.8; range = 33–92) and 84 (58.7%) of them were men. We did not find any statistically significant differences in baseline characteristics between the two study groups (Table 1).

### 3.2. Primary Endpoint: Clinical Complications after Intestinal Anastomosis

Up to day 30, 41 (28.7%) patients had postoperative complications, but we obtained no statistical differences between the two study groups (18 (25%) patients in the OHN supplement group vs. 23 (32.4%) in the IN-supplement group; *p* = 0.328; Table 2). The severities of complications according to Clavien–Dindo criteria are shown in Table 2.

The non-inferiority analysis of postoperative complications (F-M Score: *p* = 0.36; M-N Score: *p* = 0.37) between the two types of supplement was inconclusive (Table 3).

### 3.3. Secondary Endpoint: Safety Analysis of OHN versus IN Supplement

We did not find any significant differences in the number of patients who had any infections at the surgical site (*p* = 0.420) or were readmitted to the hospital (*p* = 0.312) between the two study groups. One (0.7%) surgery-associated death was reported during the study (Table 4). The mean hospital stay length was not significantly higher for patients in the IN-supplement group compared with the OHN supplement group (7.1 vs. 6.4 days, respectively; *p* = 0.287).

We showed that the OHN was not inferior to the IN supplement in terms of readmissions (F-M Score: *p* < 0.001; M-N Score: *p* = 0.01) and hospital stays of seven days or less (F-M Score: *p* < 0.001; M-N Score: *p* = 0.02) within 30 days after the surgery (Table 3). The non-inferiority analysis of surgical site infections (F-M Score: *p* = 0.15; M-N Score: *p* = 0.16) was not conclusive (Table 3).

### 3.4. Tolerance to Oral Dietary Supplement and Blood Parameters

The mean number of patients who achieved complete tolerance to both types of oral supplement was 89 (62.2%). Of them, 45 (62.5%) and 44 (62.0%) patients who were administered OHN and IN supplement showed total tolerance during the five consecutive days after the surgery, respectively. No significant differences in tolerance to both types of supplement were found (Table 5).

Blood parameters did not differ significantly between the OHN and IN supplement at each time point except for total protein mean levels at diagnosis (6.8, SD = 0.6 vs. 7.0; SD = 0.5, respectively; *p* = 0.013) (Figure S1).

## 4. Discussion

The study was aimed at comparing the efficacy and safety of postsurgical administration of OHN and IN supplement to colorectal cancer patients who arrive for surgery with normal nutritional status.

The study was based on the ERAS intervention protocol to homogenize the perioperative care for patients undergoing elective surgery. MMRH programs reduce hospital stay and complications and minimize readmission. In the meta-analysis by Greco et al., ERAS was associated with a reduction in general morbidity (relative risk (RR) = 0.60 (95% CI: 0.46–0.76) and non-surgical complications (RR = 0.40 (95% confidence interval (CI): 0.27–0.61) [20]. Moreover, nutritional interventions are important components of these programs [21,22].

While evaluating dietary supplements’ efficacy in patient recovery after surgery, we did not find any significant differences between both modalities. Therefore, immunonutrients did not add any benefits to the postoperative diet. Overall, postoperative complications were reported in 28.7% of study participants, which was correlated to other previous studies [7,23]. In a prospective cohort study published by Yeung and colleagues, elective colorectal surgery patients received either ERAS or conventional care. In the ERAS group, up to 32% of patients had complications after the intervention [23]. Another randomized trial by Moya et al. included patients treated according to ERAS and nutritional supplementation for seven days before the surgery and five days immediately after it. Although the percentage of the study population with postoperative morbidity (29%) was similar to our study, immunonutrient-enriched supplement significantly reduced the number of postoperative complications compared with the control (hypercaloric hyperproteic supplement) group (23% vs. 35.2%, respectively; *p* = 0.035) [7]. Another difference with our study was the higher number (66%) of low-grade (Clavien-Dindo grades I and II [18]) clinical complications in contrast to our study (56%).

We analyzed surgical site infections as they are the most common postoperative complication. The overall percentage found in our study (11.9%) was also comparable to that found by Moya et al. (12.6%) [7]. However, they found that the incidence of surgical site infections could be significantly reduced to 5.7% with IN supplement [7], whereas we did not find any statistically significant differences between study groups. Conversely, in the randomized trial by Burden et al. on preoperative dietary advice and oral nutritional supplements, the percentage of colorectal cancer patients with surgical site infections (28%) was much higher, and this is probably due to the inclusion of patients with weight loss and, thereby, with less probability of achieving normal nutritional status [24]. Finally, another previous study by the same authors with an identical study design found a 22% wound infection rate and no significant differences between the group of patients receiving nutrition counseling and supplement in contrast to patients with dietary counseling alone [25].

OHN supplementation was not inferior to immunonutrients regarding hospital readmissions and <7 day-stays. We found that 30-day readmission rates greatly vary across available reports, from 3.3% in randomized trials [7,26] to 6.3% in our study and 8.7% in the prospective cohort study mentioned above [23]. The highest ones were reported by Gillis et al. while studying the impact of dietary advice with whey protein on preoperative functional walking capacity and the postoperative recovery of patients subject to colorectal resection for cancer [26]. In contrast to it, the mean hospital stay length calculated in our study population (between 6.4 and 7.1 days) was consistent with that described by Yeung et al. for patients following the ERAS protocol (6.5 days) [23] and slightly above the mean length of stay reported by Gillis et al. (4.5 days) and Thornblade et al. (5.9 and 5.8 days for patients receiving or not immunonutrition before elective colorectal resection, respectively).

Approximately one-third of our study population did not completely tolerate oral supplement. We believe this is a non-negligible proportion of patients and it is in line with what we have observed in daily routine. The other two thirds of patients tolerated the 400 mL daily dose, but these results are somehow difficult to compare with other studies since adequate intake measurements utilized were very disparate. Yeung and collaborators considered the proportion of patients who took any amount of supplement when offered, which accounted for 68%, 72%, and 58% of patients in the ERAS group on days 1, 2, and 3 after surgery, respectively [23]. In contrast, Moya et al. analyzed the percentage of patients presenting good (63.1%) and poor (27.5%) tolerance and no supplement intake (9.4%), although reference intakes were not described [7].

Blood test results showed that hemoglobin, total protein, albumin, prealbumin, and transferrin levels decreased after five days and in both study arms, which may be a consequence of the high energy expenditure associated with the surgery. Similarly, in the analysis carried out by Moya et al., the authors reported a decrease in these blood parameters with no significant differences between the study groups. Nevertheless, the mean values were slightly higher than ours, which may be explained by the shorter postoperative period between the day of the surgery and the blood test in comparison to our study design (three vs. five days, respectively). Finally, blood protein levels may be a surrogate marker for nitrogen absorption capacity. Since OHN supplement contained half the amount of protein compared with immunonutrition and total protein, albumin, and prealbumin mean values were comparable in both study groups at postoperative day 5, we can speculate on an optimization (around 2-fold) in protein absorption associated with OHN supplements, which is in line with previous studies with the same supplement [27,28].

Another study investigated the absorption of protein in patients given oligomeric enteral nutrition to treat chemotherapy-related diarrhea, and they found that this was effective after eight weeks in 68.5% of cases, regardless of the type of tumor, treatment (curative/palliative, chemotherapy/radiotherapy, cytotoxic/non-cytotoxic, and targeted/non-targeted) and resectability. Of them, 48.3% showed an improvement in the nutritional status and 20.1% remained at low risk of malnutrition. Moreover, the frequency and consistency of stools were also improved even in persistent cases. Interestingly, the change in nutritional status did not depend on the increase in stool consistency; thus, a diet may enhance nutrient intake even when absorption capacity is impaired [27].

A limitation of the study was the COVID-19 pandemic, which decreased the number of surgeries during this period, thereby slowing the rate of patient inclusion and delaying the expected date of completion of the study. However, its strengths rely on the randomized and double-blind study design to minimize the biased and incorrect analysis of the supplement’s effect.

## 5. Conclusions

In MMRH programs, the occurrence of clinical complications and surgical site infections as well as hospital admissions, hospital stay, and tolerance to supplements did not differ between normo-nourished colorectal cancer patients treated with OHN and IN supplement after of surgery. Moreover, OHN may enhance protein absorption in these patients.

Hence, postoperative immunonutrients did not add any benefits to the postoperative diet and may not be necessary for patients who arrive for surgery with normal nutritional status. Future studies will confirm the efficacy of postoperative diets without IN supplementation.

## Figures and Tables

**Table 1:** Baseline patient characteristics according to both study groups.

Variable	OHN Supplement Group (n = 72)	IN Supplement Group (n = 71)	*p* Value
Age (years): Mean ± SD	69.8 ± 11.7	69.6 ± 11.8	0.657
Sex (men): n (%)	39 (54.2%)	45 (63.4%)	0.263
Previous diagnosis of diverticular disease, n (%)	11 (15.3%)	7 (9.9%)	0.329
Diabetes mellitus, n (%)	11 (15.3%)	18 (25.4%)	0.134
Smoking habit, n (%)	17 (23.6%)	15 (21.1%)	0.722
Kidney failure, n (%)	2 (2.8%)	5 (7.0%)	0.237
Previous abdominal surgery	29 (40.3%)	25 (35.2%)	0.532
Obesity (BMI > 30)	18 (25.0%)	15 (21.1%)	0.583
ASA III	20 (27.8%)	20 (28.2%)	0.958
Laparoscopic approach	55 (76.4%)	59 (83.1%)	0.604

ASA: American Society of Anesthesiologists staging system; BMI: body mass index; IN: immunonutritional; MST: malnutrition screening tool scale; OHN: oligomeric hyperproteic normocaloric; SD: standard deviation.

**Table 2:** Clinical complications within 30 days after surgery.

Variable	Occurrence/Grade	Total (n = 143)	OHN Supplement Group (n = 72)	IN Supplement Group (n = 71)	*p* Value
Complications, n (%)	Yes	41 (28.7%)	18 (25.0%)	23 (32.4%)	0.328
	No	102 (71.3%)	54 (75.0%)	48 (67.6%)	
Severity of complications ^1^, n (%)	I	7 (4.9%)	5 (6.9%)	2 (2.8%)	ND
	II	16 (11.2%)	5 (6.9%)	11 (15.5%)	
	IIIA	6 (4.2%)	3 (4.2%)	3 (4.2%)	
	IIIB	9 (6.3%)	4 (5.6%)	5 (7.0%)	
	IVA	2 (1.4%)	0 (0.0%)	2 (2.8%)	
	IVB	---	---	---	
	V	1 (0.7%)	1 (1.4%)	0 (0.0%)	

^1^ According to Clavien–Dindo classification [18]. IN: immunonutritional; ND: not determined; OHN: oligomeric hyperproteic normocaloric.

**Table 3:** Non-inferiority analysis of the difference in proportions of patients regarding the primary and secondary outcome measures.

Variable	Test	P1 (OHN)	P2 (IN)	Difference in Proportions (P1-P2)	Statistical Test Score	Probability Level	Rejection of Null Hypothesis? ^1^
Postoperative complications	F-M Score	0.75	0.68	0.07	−0.35	0.36	No
	M-N Score	0.75	0.68	0.07	−0.34	0.37	No
Surgical site infections	F-M Score	0.90	0.86	0.04	−1.02	0.15	No
	M-N Score	0.90	0.86	0.04	−1.01	0.16	No
Hospital readmissions	F-M Score	0.92	0.96	−0.04	−0.89	0.00	Yes
	M-N Score	0.92	0.96	−0.04	−0.88	0.00	Yes
<7 days hospital stays	F-M Score	0.33	0.41	−0.08	−2.17	0.01	Yes
	M-N Score	0.33	0.41	−0.08	−2.17	0.02	Yes

^1^ Null hypothesis (H0): P1 - P2 ≥ 0.1 vs. alternative hypothesis (H1): P1 - P2 < 0.1. Type I error (α) = 0.05. F-M: Farrington–Manning; IN: immunonutritional; M-N: Miettinen–Nurminen; OHN: oligomeric hyperproteic normocaloric.

**Table 4:** Surgical site infections, hospital readmissions and surgery-associated deaths within 30 days after the procedure.

Variable	Occurrence	Total (n = 143)	OHN Supplement Group (n = 72)	IN Supplement Group (n = 71)	*p* Value
Surgical site infections, n (%)	Yes	17 (11.9%)	7 (9.7%)	10 (14.1%)	0.420
	No	126 (88.1%)	65 (90.3%)	61 (85.9%)	
Readmissions, n (%)	Yes	9 (6.3%)	6 (8.3%)	3 (4.2%)	0.312
	No	134 (93.7%)	66 (91.7%)	68 (95.8%)	
Deaths, n (%)	Yes	1 (0.7%)	1 (1.4%)	0 (0.0%)	ND
	No	142 (99.3%)	71 (98.6%)	71 (100.0%)	

IN: immunonutrients; ND: not determined; OHN: oligomeric hyperproteic normocaloric.

**Table 5:** Tolerance to OHN and IN oral supplement during five days after surgery.

	OHN Supplement Group (n = 72)			IN Supplement Group (n = 71)			
Postoperative Day	Compl. Tolerance (400 mL)	Part. Tolerance (200 mL)	No Tolerance (<200 mL)	Compl. Tolerance (400 mL)	Part. Tolerance (200 mL)	No Tolerance (<200 mL)	*p* Value
1	45 (62.5%)	18 (25.0%)	9 (12.5%)	41 (57.7%)	18 (25.4%)	12 (16.9%)	0.738
2	45 (62.5%)	12 (16.7%)	15 (20.8%)	46 (64.8%)	13 (18.3%)	12 (16.9%)	0.828
3	40 (55.6%)	10 (13.9%)	22 (30.6%)	43 (60.6%)	11 (15.5%)	17 (23.9%)	0.674
4	44 (61.1%)	11 (15.3%)	17 (23.6%)	43 (60.6%)	11 (15.4%)	17 (23.4%)	0.998
5	51 (70.8%)	7 (9.7%)	14 (19.4%)	45 (63.4%)	10 (14.1%)	16 (22.5%)	0.597

Compl. tolerance: complete tolerance; IN: immunonutritional; OHN: oligomeric hyperproteic normocaloric; Part. tolerance: partial tolerance.

## Data Availability

Not applicable.

## Supplementary Materials

The following supporting information can be downloaded at: https://www.mdpi.com/article/10.3390/nu14153062/s1; Figure S1: Analytical (a) and nutritional (b) mean blood values of patients under postoperative diet with oligomeric hyperproteic normocaloric (OHN) and immunonutritional (IN) supplement. * *p* < 0.05.

## References

[1] Kreymann K.G. (2008). Early Nutrition Support in Critical Care: A European Perspective. Curr. Opin. Clin. Nutr. Metab. Care.

[2] Mariette C. (2015). Immunonutrition. J. Visc. Surg..

[3] Wanden-Berghe C., Sanz-Valero J., Arroyo-Sebastián A., Cheikh-Moussa K., Moya-Forcen P. (2016). Effects of a Nutritional Intervention in a Fast-Track Program for a Colorectal Cancer Surgery: Systematic Review. Nutr. Hosp..

[4] Skipworth R.J.E., Fearon K.C.H. (2007). The Scientific Rationale for Optimizing Nutritional Support in Cancer. Eur. J. Gastroenterol. Hepatol..

[5] Finco C., Magnanini P., Sarzo G., Vecchiato M., Luongo B., Savastano S., Bortoliero M., Barison P., Merigliano S. (2007). Prospective Randomized Study on Perioperative Enteral Immunonutrition in Laparoscopic Colorectal Surgery. Surg. Endosc..

[6] Reece L., Hogan S., Allman-Farinelli M., Carey S. (2020). Oral Nutrition Interventions in Patients Undergoing Gastrointestinal Surgery for Cancer: A Systematic Literature Review. Support. Care Cancer.

[7] Moya P., Soriano-Irigaray L., Ramirez J.M., Garcea A., Blasco O., Blanco F.J., Brugiotti C., Miranda E., Arroyo A. (2016). Perioperative Standard Oral Nutrition Supplements Versus Immunonutrition in Patients Undergoing Colorectal Resection in an Enhanced Recovery (ERAS) Protocol: A Multicenter Randomized Clinical Trial (SONVI Study). Medicine.

[8] Thornblade L.W., Varghese T.K., Shi X., Johnson E.K., Bastawrous A., Billingham R.P., Thirlby R., Fichera A., Flum D.R. (2017). Preoperative Immunonutrition and Elective Colorectal Resection Outcomes. Dis. Colon Rectum.

[9] Rice T.W. (2014). Immunonutrition in Critical Illness: Limited Benefit, Potential Harm. JAMA.

[10] Weimann A., Braga M., Carli F., Higashiguchi T., Hübner M., Klek S., Laviano A., Ljungqvist O., Lobo D.N., Martindale R. (2017). ESPEN Guideline: Clinical Nutrition in Surgery. Clin. Nutr..

[11] Van Zanten A.R.H., Sztark F., Kaisers U.X., Zielmann S., Felbinger T.W., Sablotzki A.R., De Waele J.J., Timsit J.F., Honing M.L.H., Keh D. (2014). High-Protein Enteral Nutrition Enriched with Immune-Modulating Nutrients vs Standard High-Protein Enteral Nutrition and Nosocomial Infections in the ICU: A Randomized Clinical Trial. JAMA.

[12] Moher D., Hopewell S., Schulz K.F., Montori V., Gøtzsche P.C., Devereaux P.J., Elbourne D., Egger M., Altman D.G. (2010). CONSORT 2010 Explanation and Elaboration: Updated Guidelines for Reporting Parallel Group Randomised Trials. Int. J. Surg..

[13] Ortiz Hurtado H., Asociación Española de Cirujanos (2012). Colorectal Surgery.

[14] Wittekind C., Asamura H., Sobin L.H. (2014). TNM Atlas: Illustrated Guide to the TNM Classification of Malignant Tumours.

[15] Mueller C., Compher C., Ellen D.M. (2011). A.S.P.E.N. Clinical Guidelines: Nutrition Screening, Assessment, and Intervention in Adults. J. Parenter. Enteral Nutr..

[16] Fearon K.C.H., Ljungqvist O., Von Meyenfeldt M., Revhaug A., Dejong C.H.C., Lassen K., Nygren J., Hausel J., Soop M., Andersen J. (2005). Enhanced Recovery after Surgery: A Consensus Review of Clinical Care for Patients Undergoing Colonic Resection. Clin. Nutr..

[17] American Society of Anesthesiologists (ASA) (2014). ASA Physical Status Classification System. https://www.asahq.org/standards-and-guidelines/asa-physical-status-classification-system.

[18] Dindo D., Demartines N., Clavien P.A. (2004). Classification of Surgical Complications: A New Proposal with Evaluation in a Cohort of 6336 Patients and Results of a Survey. Ann. Surg..

[19] Berriós-Torres S.I., Umscheid C.A., Bratzler D.W., Leas B., Stone E.C., Kelz R.R., Reinke C.E., Morgan S., Solomkin J.S., Mazuski J.E. (2017). Centers for Disease Control and Prevention Guideline for the Prevention of Surgical Site Infection, 2017. JAMA Surg..

[20] Greco M., Capretti G., Beretta L., Gemma M., Pecorelli N., Braga M. (2014). Enhanced Recovery Program in Colorectal Surgery: A Meta-Analysis of Randomized Controlled Trials. World J. Surg..

[21] Colebatch E., Lockwood C. (2020). Enhanced Perioperative Nutritional Care for Patients Undergoing Elective Colorectal Surgery at Calvary North Adelaide Hospital: A Best Practice Implementation Project. JBI Evid. Synth..

[22] Gustafsson U.O., Scott M.J., Schwenk W., Demartines N., Roulin D., Francis N., McNaught C.E., MacFie J., Liberman A.S., Soop M. (2012). Guidelines for Perioperative Care in Elective Colonic Surgery: Enhanced Recovery After Surgery (ERAS^®^) Society Recommendations. Clin. Nutr..

[23] Yeung S.E., Hilkewich L., Gillis C., Heine J.A., Fenton T.R. (2017). Protein Intakes Are Associated with Reduced Length of Stay: A Comparison between Enhanced Recovery After Surgery (ERAS) and Conventional Care after Elective Colorectal Surgery. Am. J. Clin. Nutr..

[24] Burden S.T., Gibson D.J., Lal S., Hill J., Pilling M., Soop M., Ramesh A., Todd C. (2017). Pre-Operative Oral Nutritional Supplementation with Dietary Advice versus Dietary Advice Alone in Weight-Losing Patients with Colorectal Cancer: Single-Blind Randomized Controlled Trial. J. Cachexia Sarcopenia Muscle.

[25] Burden S.T., Hill J., Shaffer J.L., Campbell M., Todd C. (2011). An Unblinded Randomised Controlled Trial of Preoperative Oral Supplements in Colorectal Cancer Patients. J. Hum. Nutr. Diet..

[26] Gillis C., Loiselle S.E., Fiore J.F., Awasthi R., Wykes L., Liberman A.S., Stein B., Charlebois P., Carli F. (2016). Prehabilitation with Whey Protein Supplementation on Perioperative Functional Exercise Capacity in Patients Undergoing Colorectal Resection for Cancer: A Pilot Double-Blinded Randomized Placebo-Controlled Trial. J. Acad. Nutr. Diet..

[27] Sanz-Paris A., Martinez-Trufero J., Lambea-Sorrosal J., Calvo-Gracia F., Milà-Villarroel R. (2020). Clinical and Nutritional Effectiveness of a Nutritional Protocol with Oligomeric Enteral Nutrition in Patients with Oncology Treatment-Related Diarrhea. Nutrients.

[28] Ferreiro B., Llopis-Salinero S., Lardies B., Granados-Colomina C., Milà-Villarroel R. (2021). Clinical and Nutritional Impact of a Semi-Elemental Hydrolyzed Whey Protein Diet in Patients with Active Crohn’s Disease: A Prospective Observational Study. Nutrients.

