# Supplementary material for: Postoperative Diet with an Oligomeric Hyperproteic Normocaloric Supplement versus a Supplement with Immunonutrients in Colorectal Cancer Surgery: Results of a Multicenter, Double-Blind, Randomized Clinical Trial
Source: Nutrients. 2022 Jul 26;14(15):3062. doi: 10.3390/nu14153062 (PMC9331223; doi:10.3390/nu14153062)
Supplement: Supplementary file 1 [file nutrients-14-03062-s001.zip › nutrients-1790027-supplementary.pptx]

## Slide 1
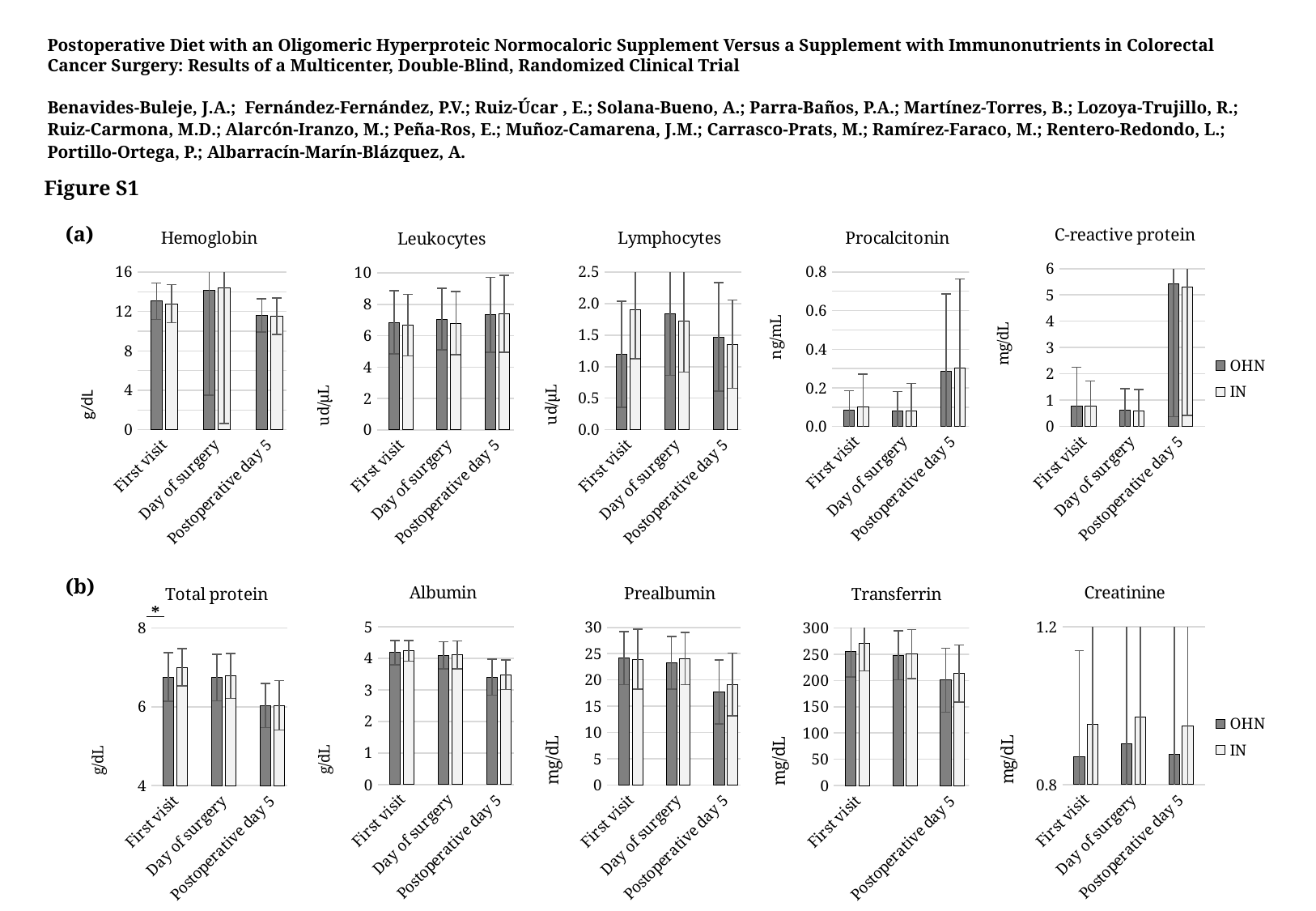

Postoperative Diet with an Oligomeric Hyperproteic Normocaloric Supplement Versus a Supplement with Immunonutrients in Colorectal Cancer Surgery: Results of a Multicenter, Double-Blind, Randomized Clinical Trial
Benavides-Buleje, J.A.; Fernández-Fernández, P.V.; Ruiz-Úcar , E.; Solana-Bueno, A.; Parra-Baños, P.A.; Martínez-Torres, B.; Lozoya-Trujillo, R.; Ruiz-Carmona, M.D.; Alarcón-Iranzo, M.; Peña-Ros, E.; Muñoz-Camarena, J.M.; Carrasco-Prats, M.; Ramírez-Faraco, M.; Rentero-Redondo, L.; Portillo-Ortega, P.; Albarracín-Marín-Blázquez, A.
Figure S1
### Chart: C-reactive protein
| Category | OHN | IN |
|---|---|---|
| First visit | 0.761 | 0.785 |
| Day of surgery | 0.627 | 0.577 |
| Postoperative day 5 | 5.433 | 5.313 |
### Chart: Hemoglobin
| Category | OHN | IN |
|---|---|---|
| First visit | 13.036 | 12.787 |
| Day of surgery | 14.137 | 14.411 |
| Postoperative day 5 | 11.604 | 11.508 |
### Chart: Procalcitonin
| Category | OHN | IN |
|---|---|---|
| First visit | 0.084 | 0.102 |
| Day of surgery | 0.08 | 0.082 |
| Postoperative day 5 | 0.287 | 0.303 |
### Chart: Lymphocytes
| Category | OHN | IN |
|---|---|---|
| First visit | 1.198 | 1.907 |
| Day of surgery | 1.835 | 1.722 |
| Postoperative day 5 | 1.47 | 1.354 |
### Chart: Leukocytes
| Category | OHN | IN |
|---|---|---|
| First visit | 6.85 | 6.677 |
| Day of surgery | 7.056 | 6.8 |
| Postoperative day 5 | 7.335 | 7.39 |(a)
### Chart: Creatinine
| Category | OHN | IN |
|---|---|---|
| First visit | 0.87 | 0.953 |
| Day of surgery | 0.903 | 0.972 |
| Postoperative day 5 | 0.877 | 0.948 |
### Chart: Albumin
| Category | OHN | IN |
|---|---|---|
| First visit | 4.182 | 4.242 |
| Day of surgery | 4.098 | 4.111 |
| Postoperative day 5 | 3.407 | 3.486 |
### Chart: Prealbumin
| Category | OHN | IN |
|---|---|---|
| First visit | 24.14 | 23.954 |
| Day of surgery | 23.291 | 24.089 |
| Postoperative day 5 | 17.716 | 19.126 |
### Chart: Total protein
| Category | OHN | IN |
|---|---|---|
| First visit | 6.756 | 7.0 |
| Day of surgery | 6.744 | 6.779 |
| Postoperative day 5 | 6.031 | 6.037 |
### Chart: Transferrin
| Category | OHN | IN |
|---|---|---|
| First visit | 255.27 | 271.18 |
| Day of surgery | 248.17 | 249.95 |
| Postoperative day 5 | 200.84 | 213.45 |(b)
(b)
*
